# Analyzing EEG signals to detect unexpected obstacles during walking

**DOI:** 10.1186/s12984-015-0095-4

**Published:** 2015-11-14

**Authors:** R. Salazar-Varas, Á. Costa, E. Iáñez, A. Úbeda, E. Hortal, J. M. Azorín

**Affiliations:** Center for Research and Advanced Studies (Cinvestav), Parque de Investigación e Innovación Tecnológica km 9.5 de la Autopista Nueva al Aeropuerto, 201., Monterrey, 66600 NL Mexico; Brain-Machine Interface Systems Lab, Universidad Miguel Hernández de Elche, Av. de la Universidad, S/N, Elche, 03202 Spain

**Keywords:** BCI, Obstacle detection, Lower limb rehabilitation, EEG, Wearable robot, Exoskeleton

## Abstract

**Background:**

When an unexpected perturbation in the environment occurs, the subsequent alertness state may cause a brain activation responding to that perturbation which can be detected and employed by a Brain-Computer Interface (BCI). In this work, the possibility of detecting a sudden obstacle appearance analyzing electroencephalographic (EEG) signals is assessed. For this purpose, different features of EEG signals are evaluated during the appearance of sudden obstacles while a subject is walking on a treadmill. The future goal is to use this procedure to detect any obstacle appearance during walking when the user is wearing a lower limb exoskeleton in order to generate an emergency stop command for the exoskeleton. This would enhance the user-exoskeleton interaction, improving the safety mechanisms of current exoskeletons.

**Methods:**

In order to detect the change in the brain activity when an obstacle suddenly appears, different features of EEG signals are evaluated using the recordings of five healthy subjects. Since the change in the brain activity occurs in the time domain, the features evaluated are: common spatial patterns, average power, slope, and the coefficients of a polynomial fit. A Linear Discriminant Analysis-based classifier is used to differentiate between two conditions: the appearance or not of an obstacle. The evaluation of the performance to detect the obstacles is made in terms of accuracy, true positive (TP) and false positive (FP) rates.

**Results:**

From the offline analysis, the best performance is achieved when the slope or the polynomial coefficients are used as features, with average detection accuracy rates of 74.0 and 79.5 %, respectively. These results are consistent with the pseudo-online results, where a complete EEG recording is segmented into windows of 500 ms and overlapped 400 ms, and a decision about the obstacle appearance is made for each window. The results of the best subject were 11 out of 14 obstacles detected with a rate of 9.09 FPs/min, and 10 out of 14 obstacles detected with a rate of 6.34 FPs/min using slope and polynomial coefficients features, respectively.

**Conclusions:**

An EEG-based BCI can be developed to detect the appearance of unexpected obstacles. The average accuracy achieved is 79.5 % of success rate with a low number of false detections. Thus, the online performance of the BCI would be suitable for commanding in a safely way a lower limb exoskeleton during walking.

## Background

Brain activity can be classified as evoked or spontaneous depending on the volitional capability of the user to control it. For instance, the performance of real and imaginary movements induces changes in the brain activity that can be controlled by the user, while the perception of a visual, auditory or sensitive stimulus provokes automatic changes in the brain potentials, called event-related potentials (ERP) [1]. Some ERPs are related to an alertness state like contingent-negative variation (CNV) [2]. It occurs when a warning stimulus (W1) appears followed by an imperative stimulus (W2) that requires a mental or motor response. When the interval between W1 and W2 is more than two seconds it is possible to distinguish two CNV components: 1) an early component registered over the fronto-central area with bigger amplitude between 550 and 700 ms after the warning stimulus W1, and 2) the late component which occurs in a centro-parietal location with its maximum amplitude occurring 200 ms before the imperative stimulus W2. The early CNV reflects the processing of the warning signal and the anticipation of the upcoming event, the late CNV involves neural activity prior to reaction [3, 4].

These changes in the brain activity, which can be recorded in a non-invasive way through electroencephalography (EEG), can be used to develop a Brain-Computer Interface (BCI) to establish a communication pathway between a subject and a device without the use of peripheral nerves [5, 6]. Until the last decade, BCI applications in the rehabilitation field were limited to the control of upper limb prosthetics and orthotics [7, 8]. More recently, it has been demonstrated that information about locomotion can be extracted from the primary motor cortex [9], and the first efforts to decode gait parameters from EEG recordings are being developed [10, 11]. In this sense, BCIs have started to be used in the rehabilitation of lower limbs, being used to command exoskeletons. An exoskeleton is an assistive device that helps the user in movement execution tasks [12]. Current approaches to command exoskeletons only consider a planned and voluntary control of the start and stop of the gait based on sensorimotor rhythms [13–16]. Also, brain activity has been analyzed when there are volitional changes in the gait speed [17]. The results of that work suggest that it is possible to detect a volitional change in gait speed and that the parietal cortex may be involved in motor planning. In order to command safely an exoskeleton, it would be desirable that the exoskeleton would implement a procedure to detect the sudden appearance of any obstacle. Although this procedure could be developed using a camera-based system, achieving this detection through EEG signals would imply a greater involvement of the user during the gait process. As a consequence, user-exoskeleton interaction would improve. Furthermore, if the obstacle were detected through EEG signals, the user wold be able to volitionally discriminate if the obstacle is dangerous or not.

The interaction between brain and spinal neuronal activity during the preparation and performance of obstacle avoidance has been previously investigated [18]. In that work, subjects were acoustically informed about an approaching obstacle. Subjects avoided the obstacle stepping over it with the right foot. That means the avoidance of the obstacle was always in the swing of the right leg. However, in real life the obstacle appearance is unexpected and the subjects must react immediately regardless the phase of the gait in which they are. Moreover, the obstacle cannot always be avoided.

The change of the EEG activity when an obstacle suddenly appears while a subject is walking was assessed in our prior work [19]. Different responses to the obstacle appearance were evaluated: to avoid the obstacle (subjects stop their walking), to ignore the obstacle appearance (subjects do not stop their walking), to avoid the obstacle with a delay (subjects stop their walking a few seconds after the obstacle appears), and to react without obstacle appearance (subjects stop their walking when they want to). The results obtained suggested that there is a change in the EEG potential over the fronto-central area when the subjects react to avoid the obstacle.

The goal of the present work is to evaluate if it is possible to detect the obstacle appearance from the change in the brain activity. Different features of the EEG signals are evaluated separately in order to select one that allows detecting the appearance of an unexpected obstacle during walking previous to subject’s reaction. For each feature, different time intervals are evaluated in order to obtain the optimal in terms of the performance in the discrimination task. This means, the optimal time interval is used to extract the feature to train the classifier. Not only is the BCI performance in the detection of obstacles evaluated offline, but a pseudo-online analysis is also used to evaluate the performance in similar conditions to real time. The final application will be to send a control command to stop the exoskeleton when an obstacle is detected.

This work is part of the BioMot Project (Smart Wearable Robots with Bioinspired Sensory-Motor Skills), funded by the Commission of the European Union under Grant Agreement number IFP7-ICT- 2013-10-611695 [20]. The project is focused, among others aspects, on developing a wearable robot (WR) that uses EEG information as part of the control system in order to involve the user in the rehabilitation therapy.

## Methods

### Experimental set-up

#### EEG acquisition equipment

The EEG signals are acquired using the commercial amplifier g.USBamp of the g.Tec company with the active electrodes g.LADYbird to improve signal/noise ratio. The acquisition of EEG signals is done using 32 electrodes placed over the scalp with the following distribution: Fz, FCz, FC5, FC3, FC1, FC6, FC4, FC2, Cz, C5, C3, C1, C6, C4, C2, CPz, CP5, CP3, CP1, CP6, CP4, CP2, Pz, P3, P1, P4, P2, POz, PO7, PO3, PO8 and PO4, according to the International 10/10 System. Signals are digitalized at a sampling frequency of 1200 Hz.

Due to technical complications, some registers were performed with the ActiCHamp equipment of the BrainProducts company. The same distribution of electrodes was used and the signals were registered with a sampling frequency of 500 Hz.

#### Inertial measurement units

During the tests, kinematic information is also recorded in order to know when the subject has reacted to the obstacle appearance. Seven inertial measurement units (IMUs) are located on the lower limbs, three on each limb (ankle, calf muscle and quadriceps), and one on the lumbar region. Each IMU registers 19 parameters: rotation matrix (nine parameters), acceleration matrix (three parameters, m/s^2^), angular velocity matrix (three parameters, rad/s), magnetic field (three parameters) and temperature. The sampling frequency is 30 Hz.

#### Additional equipment

To achieve a constant velocity during the gait, a treadmill Pro-form Performance 750 is used. Two procedures are used to simulate the appearance of an obstacle during walking on the treadmill. In the first one, a line laser is projected over the treadmill to simulate the appearance of the obstacle. The laser module emits a wavelength of 635 nm (red color), with an output power of 3 mW. In the second one, a screen placed in front of the treadmill changes its color to simulate the appearance of the obstacle.

### Experimental procedure

**Fig. 1:**
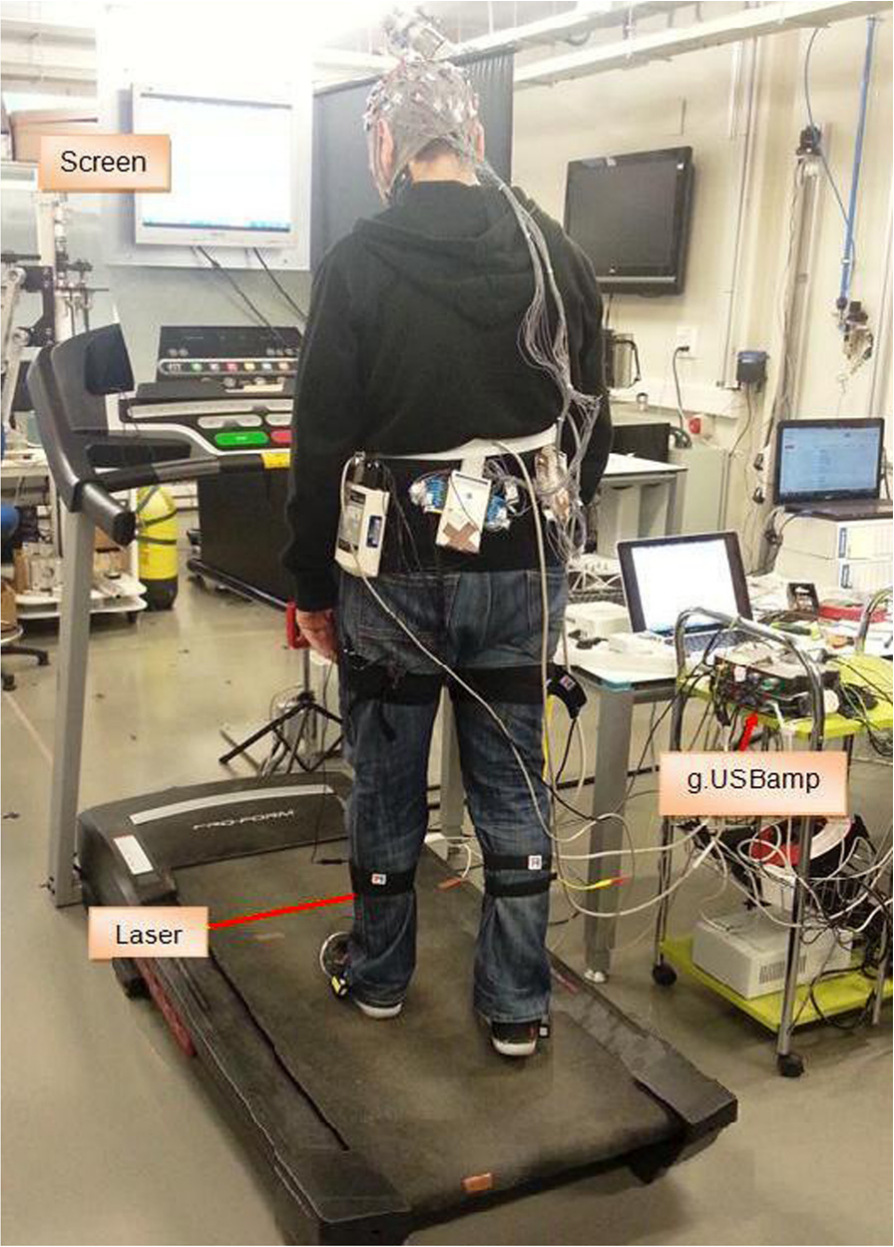
Experimental environment. The subject is walking on the treadmill while the EEG signals are recorded. For the Laser mode, a line is projected over the treadmill (using a laser) while the subject is walking. For the Screen mode, the background color of the screen placed in front of the user changes

To use the information recorded by IMUs in the EEG analysis, a synchronized register of EEG and kinematics is necessary. So, a software based on Matlab has been developed allowing the synchronized register. The software allows connecting both systems by using the Application Programming Interface (API) provided. First, a configuration of both is performed. Then the experiment starts by asking the subject to stand still on the treadmill for a few seconds while the IMUs are calibrated. Once this calibration finishes, the subject starts walking on the treadmill with a constant velocity of 2 km/h and 0 degrees of inclination. As it has been indicated previously, the obstacle appearance is simulated in two ways: 1) The projection of a line over the treadmill (using a laser) interfering with the subject’s gait (labeled as *Laser* mode); 2) The change of the background color of a screen placed in front of the treadmill (labeled as *Screen* mode). A representation of the experimental environment is shown in Fig. 1.

Different responses regarding the obstacle appearance are requested to the subject:

- *Reaction*. The subject reacts to the obstacle stopping the gait for a moment and then starts the gait again. If the subject stops the gait, the treadmill has enough space to move backwards during a short period of time.
- *Delayed reaction*. The subject continues walking two or three steps after the obstacle appearance, stops the gait for a moment and then starts the gait again.
- *No reaction*. The subject ignores the obstacle appearance and continues walking normally.
- *Free reaction*. The subject freely decides when to stop the gait several times during the recording.

In the case of *Reaction* and *No reaction*, the obstacle representation (i.e. Laser and Screen) is held during 5 s, while in the case of *Delayed* reaction is held during 10 s.

A run consists of: 180 s of *Reaction* condition, 240 s of *Delayed reaction* condition, 180 s of *No reaction* condition and 120 s of *Free reaction* condition. In all cases, the obstacle appears seven times for each obstacle representation. In order to avoid subject’s prediction of the obstacle apparition, the inter obstacle time has a random value between 2–5 s. This procedure has been tested in five male healthy subjects (labeled as S1, S2, S3, S4, S5) with ages between 24 and 29 years, all of them right handed, without any neurological disorder and with normal vision. The subjects have performed a total of four runs. The experiments were approved by the Ethics Committee of the Miguel Hernandez University of Elche (Spain). All subjects were informed and signed an informed consent according to Helsinski declaration.

The register of S1, S2 and S3 was carried out with the g.Tec, while the register of S4 and S5 was performed with the BrainProducts equipment. For these two subjects only Screen obstacle representation was used.

All four different responses were used in our previous work [19] to evaluate how the EEG signals changed in each case. However, in this work only the *Reaction* data are used since the goal is to detect the obstacle appearance from EEG signals when the subject reacts to the obstacle (actually before the subject stops their gait).

### EEG signal processing and analysis

The *Reaction* data have been analyzed offline. The channels FC5, FC6, CP5, CP6, PO3, PO4, PO7, PO8, C5 and C6 are affected by movement artifacts since they are located on the periphery of the head, and due to the cap features, there is a poor contact between the scalp and the electrodes in that area when the subject is walking. For this reason, these electrodes are discarded. Furthermore, a digital bandpass filter from 0.4 to 3 Hz is applied. This filter was selected on the basis of our previous work [19], where we found that changes in the EEG signals due the emergence of obstacles appear in low frequencies.

In order to smooth the basal brain activity contributions, the common average reference (CAR) is applied, computed as:

(1) $$ y_{i}(t)=x_{i}(t)-\frac{1}{M}\sum_{m=1}^{M}x_{m}(t),  $$

where *y*_*i*_(*t*) is the filtered signal, *x*_*m*_(*t*) is the signal recorded in the time instant *t*=1,2,…,*T* at electrode *m*=1,2,…,*M*, being *T* the total number of data points and *M* the total number of electrodes (in this work *M* = 22).

Filtered trials with standard deviations higher than 40 *μ*V in any channel were rejected.

### Feature extraction

Given an EEG recording stored in a matrix *Y*_*M*×*T*_, where *M* is the total number of EEG channels, and *T* the total number of data points, the features vector ***f*** representing the EEG recording is generated by the features extracted following the next methodologies:

- **Common Spatial Patterns (CSP).** It is a spatial filter based on the mutual diagonalization of each covariance matrix for each class to be discriminated [21]. The spatial covariance for each class is obtained averaging the covariance matrix over all trials of each class. In the case of two classes, the composite spatial covariance is given by $C_{C}=\bar {C_{1}}+\bar {C_{2}}$, which can be factorized by $C_{C}=U_{C}D_{C}{U_{C}^{T}}$ where *U*_*C*_ and *D*_*C*_ correspond to the eigenvectors and the diagonal matrix of eigenvalues, respectively. Applying the whitening transformation $P=\sqrt {D_{C}^{-1}}{U_{C}^{T}}$, the spatial covariance matrices are transformed as $S_{1}=P\bar {C_{1}}P^{T}$ and $S_{2}=P\bar {C_{2}}P^{T}$. Now *S*_1_ and *S*_2_ share common eigenvectors, i.e. *S*_1_=*BD*_1_*B*^*T*^ and *S*_2_=*BD*_2_*B*^*T*^. With the projection matrix *W*=*P*^*T*^*B*, the decomposition of a trial *Y* is given as *Z*=*WY*. Due to the calculation of W, the *c* first and *c* last rows of *Z* allow the best class discrimination and are used to obtain the feature vector [22]:
  (2) $$ \boldsymbol{\mathit{f}}=var(Zp),  $$
  where *Z*_*p*_ is the matrix containing the *c* first and *c* last rows of the original *Z* matrix.
- **Power.** The mean power of the EEG signal is obtained by:
  (3) $$ \boldsymbol{\mathit{f}}(m)=\frac{1}{T}\sum_{t=1}^{T} {y_{m}^{2}}(t),  $$
  where *y*_*m*_(*t*) is the signal recorded at electrode *m*=1,2,…,*M* at time instant *t*=1,2,3,…,*T*.
- **Slope.** Even though the slope can be computed by a polynomial fit like in [23, 24], in this work is computed as the slope of the straight line passing through the minimum voltage, *Vmin*, and the maximum voltage, *Vmax*, of the EEG signal, exploiting the fact that latencies are descriptive features of an ERP [1]. Therefore, the slope is computed as:
  (4) $$ \boldsymbol{\mathit{f}}(m)=\frac{Vmin_{m}-Vmax_{m}}{Tmin_{m}-Tmax_{m}},  $$
  where *Tmin*_*m*_ and *Tmax*_*m*_ are the data points in which *Vmin*_*m*_ and *Vmax*_*m*_ occur, respectively, in the signal recorded at electrode *m*=1,2,…,*M*.
- **Polynomial.** The coefficients of a polynomial of degree *N* which best fits the average signal over a selected brain region, in a least square sense. The feature vector is given by:
  (5) $$ \boldsymbol{\mathit{f}}=\left[ \alpha_{0}, \alpha_{1},\alpha_{2},\ldots,\alpha_{N} \right].  $$
  where *α*_0_,*α*_1_,*α*_2_,…,*α*_*N*_ are the coefficients of the polynomial.

### Classifier

A classifier based on linear discriminant analysis (LDA) has been used to differentiate between two classes from the feature vector ***f***: *Reaction* or *Normal walking*. LDA seeks to reduce the dimensionality of the data and establishes a surface decision in the features space which separates the points into two groups, each one related to one class [25]. The discrimination function can be written as:

(6) $$ {\mathrm{d}}=\boldsymbol{\mathit{v}}^{T}\boldsymbol{\mathit{f}}+{\mathrm{v_{0}}},  $$

where ***v*** is a normal vector to the surface decision and *v*_0_ is an offset value. The discrimination rule is:

(7) $$\begin{array}{@{}rcl@{}} \text{if} \quad \text{d}>0 \quad{\boldsymbol{\mathit{f}}} \quad \in \quad\text{Class1},\\ \text{otherwise}\quad {\boldsymbol{\mathit{f}}}\quad\in \quad\text{Class 2}. \end{array} $$

### Pseudo-online classification

In this classification, a full EEG recording is segmented into windows of 500 ms and overlapped 400 ms to be processed (band pass and CAR filter are applied) in order to assign each window to one class: *Reaction* or *Normal walking*. When a window coming from the *Reaction* class is assigned to that class a true positive (TP) occurs. When a window coming from the *Normal walking* class is assigned to the *Reaction* class, a false positive (FP) occurs. Since the windows are overlapped, there are redundant information generating repeated *Reaction* detections. To solve this situation, a detection is taken into account as a true detection when *k* consecutive windows are assigned to the *Reaction* class. Depending on the value of *k*, TP and FP rates behave differently. Then, it is important to perform an analysis to decide the adequate value of *k*.

## Summary of our previous work

**Fig. 2:**
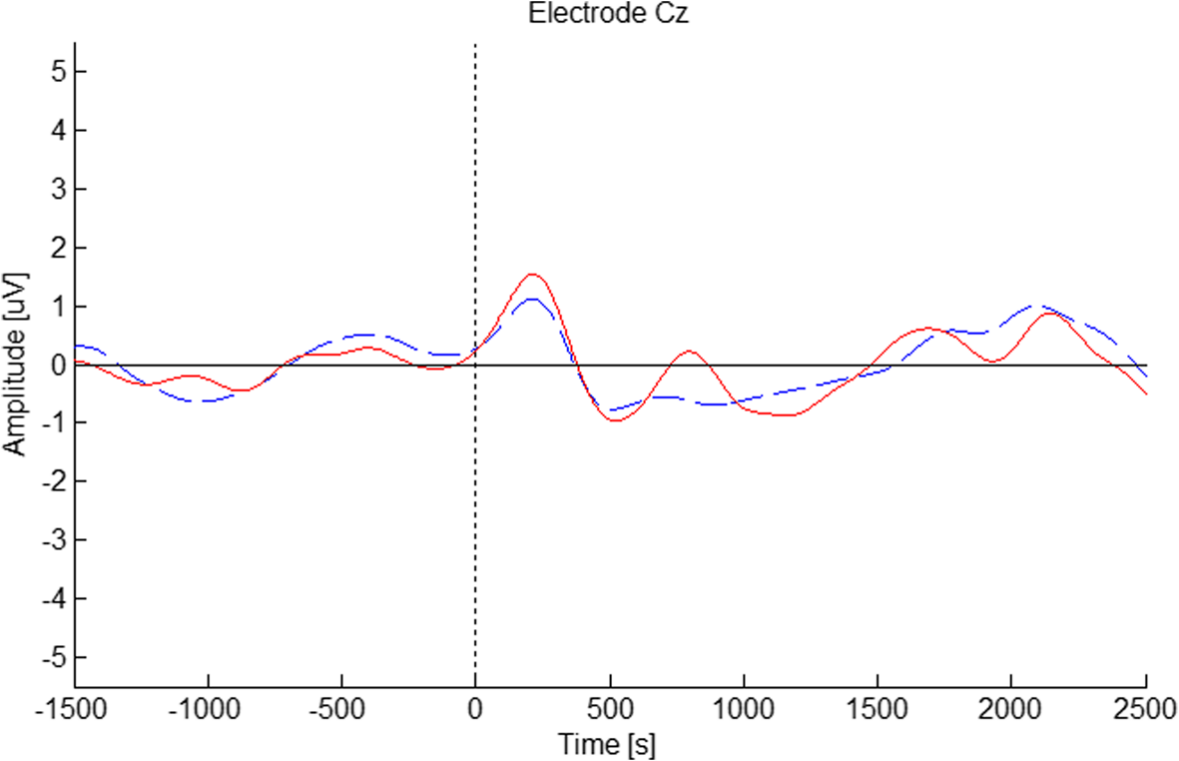
Comparison of the signals obtained by two different acquisition equipments. Graphic shows the EEG signal recorded at electrode Cz during the *Reaction* response for the Screen mode. The red line corresponds to the BrainProducts equipment (subject S4), and blue dashed line corresponds to the g.Tec equipment (subject S3). The obstacle appears at *t*=0 ms

To provide context about the EEG activity behavior when an obstacle suddenly appears during walking, the results obtained in our prior work [19] are summarized in this section. Since the signals were recorded using two different equipments, the wave form of the average potential of two different subjects (each one corresponding to one equipment) is compared in Fig. 2 during the *Reaction* response. This figure shows the signal recorded at electrode Cz since the change in the potential when the subject reacts is better observed in that electrode. As it can be seen, the signals are very similar for both equipments. Therefore, henceforward, all the graphics for the Screen representation are constructed using the data of both equipments.

**Fig. 3:**
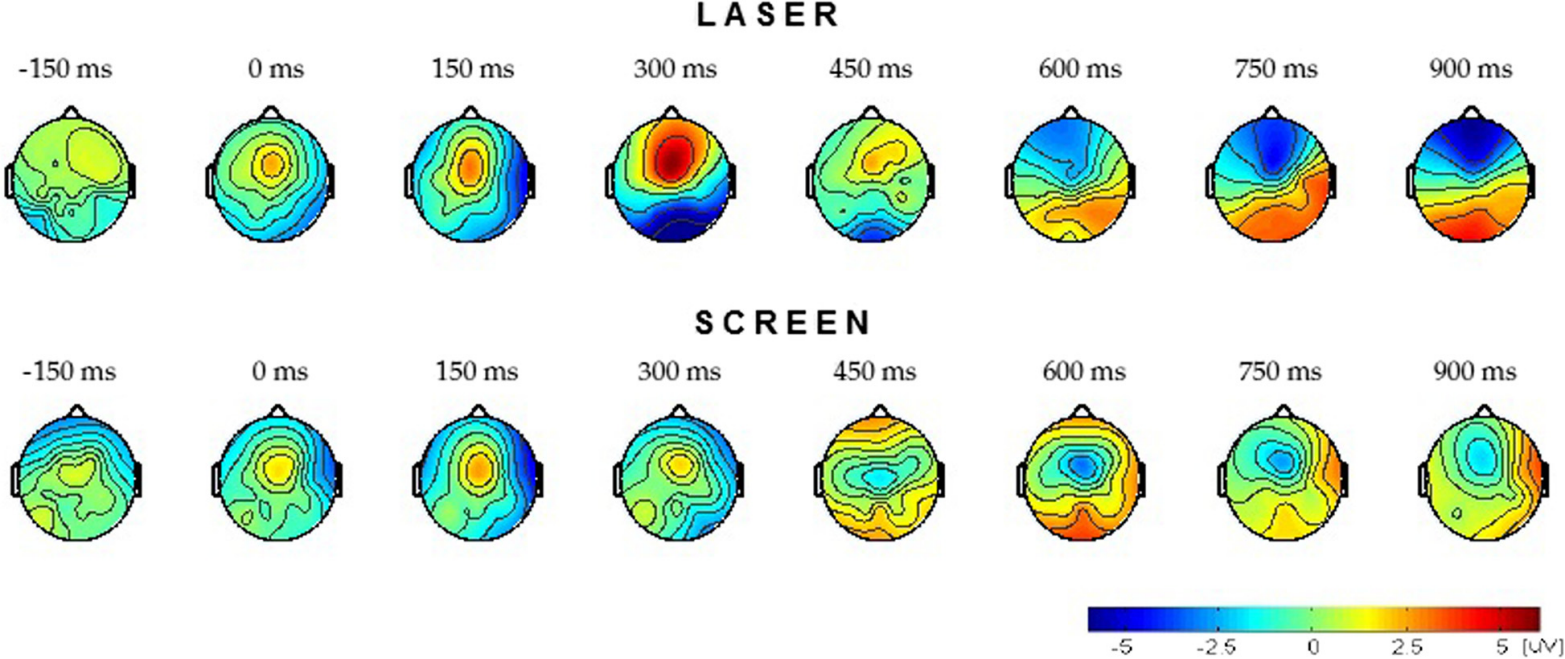
Reaction. Scalp distribution of the average potential for all subjects (three for the Laser mode and five for the Screen mode) at different time instants. *Top:* Laser mode. *Bottom:* Screen mode. The obstacle appears at *t*=0 ms

Figure 3 shows the scalp distribution of the average potential for all the subjects (three for Laser and five for Screen) at different time instants. The graphic on top corresponds to the Laser mode and the graphic on the bottom corresponds to the Screen mode. The obstacle appears at *t*=0 ms. It is possible to see a positive activity focused over the fronto-central area at *t*=300 ms for the Laser mode. The same behavior is observed focused over the central area at *t*=150 ms for the Screen mode. Later, around *t*=600 ms, this potential begins to change to negative values. This scalp distribution is consistent with the fact that a warning stimulus modifies the activity of the dorsal premotor cortex areas [26]. The described change in the brain activity occurs previous to the average response time (i.e. when the subject stops their gait to react to the obstacle), which is: 1056 ± 246 ms for the Laser mode and 1032 ± 306 ms for the Screen mode. This fact makes feasible to develop an EEG-based BCI to control a lower limb exoskeleton. This control will consist in the execution of emergency stops of the exoskeleton when an unexpected obstacle appears during walking.

**Fig. 4:**
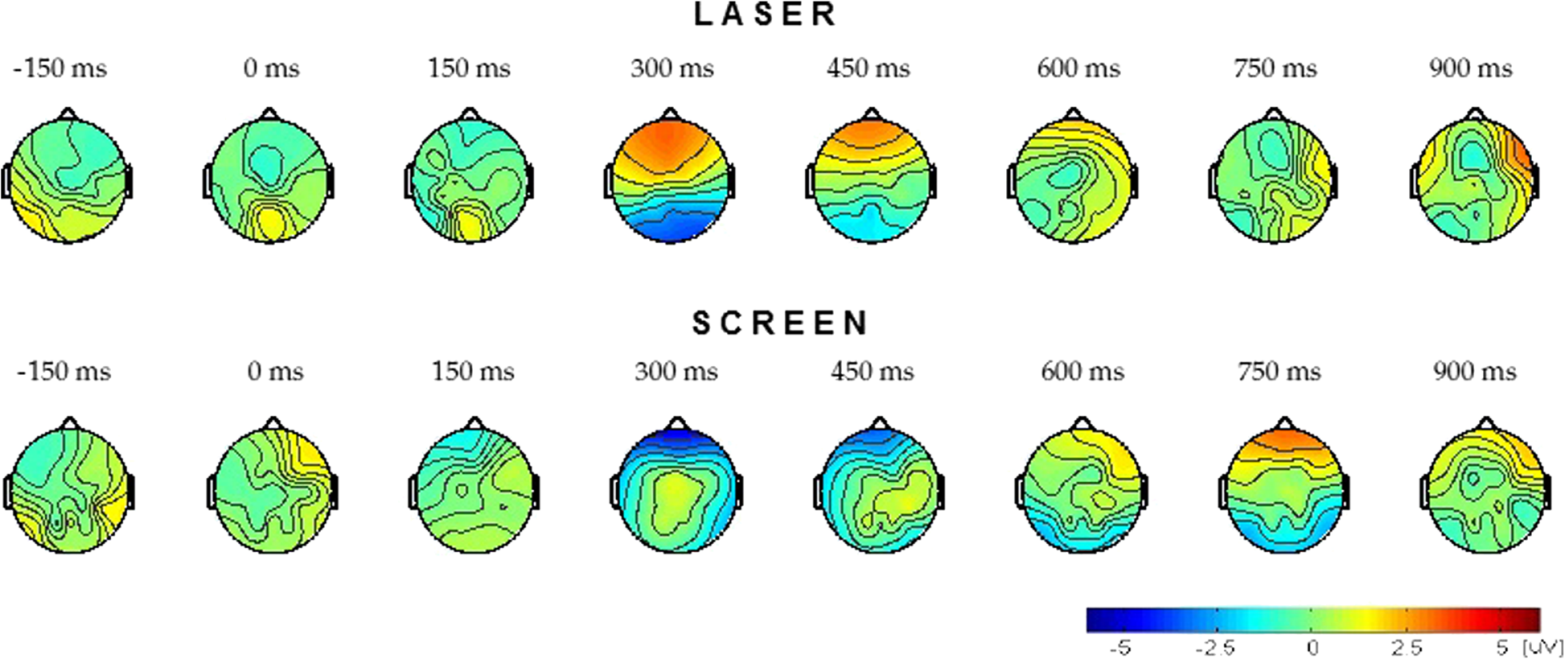
No reaction. Scalp distribution of the average potential for of all subjects (three for the Laser mode and five for the Screen mode) at different time instants. *Top:* Laser mode. *Bottom:* Screen mode. The obstacle appears at *t*=0 ms

**Fig. 5:**
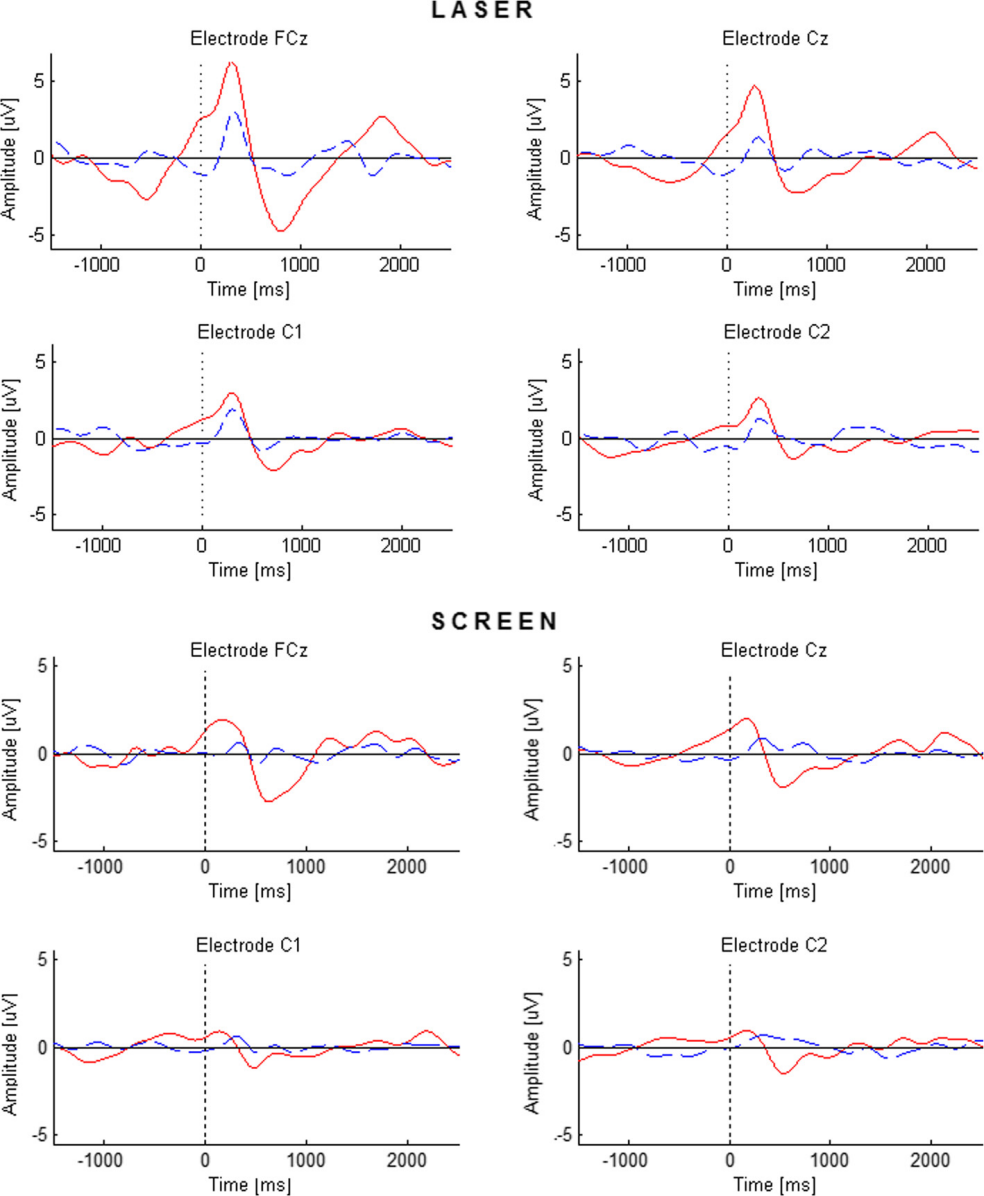
Average potential of all subjects (three for the Laser mode and five for the Screen mode) of the EEG signals recorded at electrodes FCz, Cz, C1 and C2. *Top:* Laser mode. *Bottom:* Screen mode. The obstacle appears at *t*=0 ms. The straight red line represents the *Reaction* response while the dashed blue line corresponds to the *No reaction* response

The scalp distribution of the brain activity when the subject ignores the obstacle appearance is shown in Fig. 4. A similar change is observed during the *No reaction* response for the Laser mode. However, this change does not have significant difference with the brain activity during normal walking. The statistical significance of the difference between the EEG signal recorded while the subject is walking normally and the signal obtained for the *Reaction* and *No Reaction* responses is evaluated through a significance test performed according to [27]. The theoretical justification of this method is based on the permutation test theory. The results obtained for each electrode are reported in Table 1. With a significance level *α*=0.01, it is possible to say that there is a significant difference between the EEG signals recorded while the subject is walking normally and the EEG signals recorded during the *Reaction* response at the electrodes Fz, FCz, FC1, FC2, Cz, C1 and C2 for the Laser mode, and at the electrodes FCz, Cz, C1 and C2 for the Screen mode. This fact agrees with the scalp distribution observed in Fig. 3. The average of the EEG signal recorded at the electrodes FCz, Cz, C1, and C2 (which present significant differences for both modes) is shown in Fig. 5. The continuous red line corresponds to the *Reaction* response and the dashed blue line corresponds to the *No reaction* response. As it can be seen, the brain activity corresponding to the *Reaction* response presents similar behavior for both obstacle representations, Laser and Screen, with the difference that in the Screen mode the positive activity occurs some miliseconds before than in Laser mode. Based on the results it is possible to assure that the positive deflection reflects the processing of the obstacle appearance and the negative deflection is related to the preparation to react (i.e. stop the gait) and this is the activity that concerns us in the present work. The observed behavior is similar to the early stage of CNV in which the negative deflection reflects the anticipation of the upcoming event.

**Table 1:** Results of the significance tests on waveform comparisons

Electrode	Normal vs Normal		Reaction vs Normal		No Reaction vs Normal	
aLaser	Screen	Laser	Screen	Laser	Screen	
Fz	0.002**	0.896	0.007**	0.611	0.065	0.031
FCz	0.052	0.104	0.000**	0.000**	0.000**	0.825
FC3	0.033	0.371	0.138	0.852	0.000**	0.545
FC1	0.134	0.634	0.000**	0.028	0.026	0.695
FC4	0.712	0.799	0.508	0.714	0.181	0.141
FC2	0.001**	0.588	0.001**	0.063	0.012	0.127
Cz	0.169	0.226	0.000**	0.000**	0.080	0.350
C3	0.349	0.720	0.438	0.709	0.368	0.644
C1	0.181	0.148	0.000**	0.002**	0.109	0.681
C4	0.708	0.117	0.473	0.213	0.177	0.949
C2	0.005**	0.019	0.004**	0.001**	0.253	0.407
CPz	0.103	0.429	0.543	0.824	0.268	0.013
CP3	0.289	0.648	0.229	0.400	0.782	0.052
CP1	0.355	0.764	0.016	0.246	0.289	0.589
CP4	0.092	0.669	0.006**	0.050	0.172	0.468
CP2	0.003**	0.169	0.041	0.958	0.017	0.169
Pz	0.201	0.222	0.000**	0.063	0.100	0.304
P3	0.026	0.912	0.020	0.552	0.045	0.395
P1	0.002**	0.827	0.001**	0.088	0.065	0.202
P4	0.026	0.541	0.000**	0.187	0.029	0.111
P2	0.000**	0.805	0.000**	0.016	0.693	0.141
POz	0.104	0.727	0.000**	0.045	0.210	0.762

The values which present significant difference (*p*<0.01) are marked with **

Given the similarity of the brain activity under the two representations, the data from the Laser mode and the Screen mode are considered as resulting from a common condition: Reaction to the obstacle. Therefore, two classes have to be differentiated: *Reaction* and *Normal walking*.

## Results

### Offline analysis

Since the change of the EEG activity is focussed over the fronto-central area, the features described above were extracted using the channels FC1, FCz, FC2, C1, Cz, C2, except in the case of CSP, where all the channels were used. The feature vector has: six components in the case of power and slope (one for each electrode); five in the case of polynomial fit (as the degree of the polynomial used in the fit is five); and six in the case of CSP (as *c*=3).

In order to know the best time interval to extract the features to train the system and to achieve the best discrimination, four different time intervals are evaluated. Based on the behavior of the EEG signals described above, the intervals to evaluate are: 0–500, 150–650, 300–800 and 0–800 ms, considering that the obstacle appears at *t*=0 ms. The data extracted at these time intervals are used as *Reaction* class. *Normal walking* class is made with segments of the same length, but extracted from the EEG signals recorded while the subject’s walking was normal. The total number of trials in the *Reaction* class for each subject is: 53 for subject S1, 52 for subject S2, 34 for subject S3, 58 for subject S4 and 27 for subject S5. Since in real life the *Normal walking* class is more frequent, the number of trials for this class is twice the number of trials for the *Reaction* class.

**Fig. 6:**
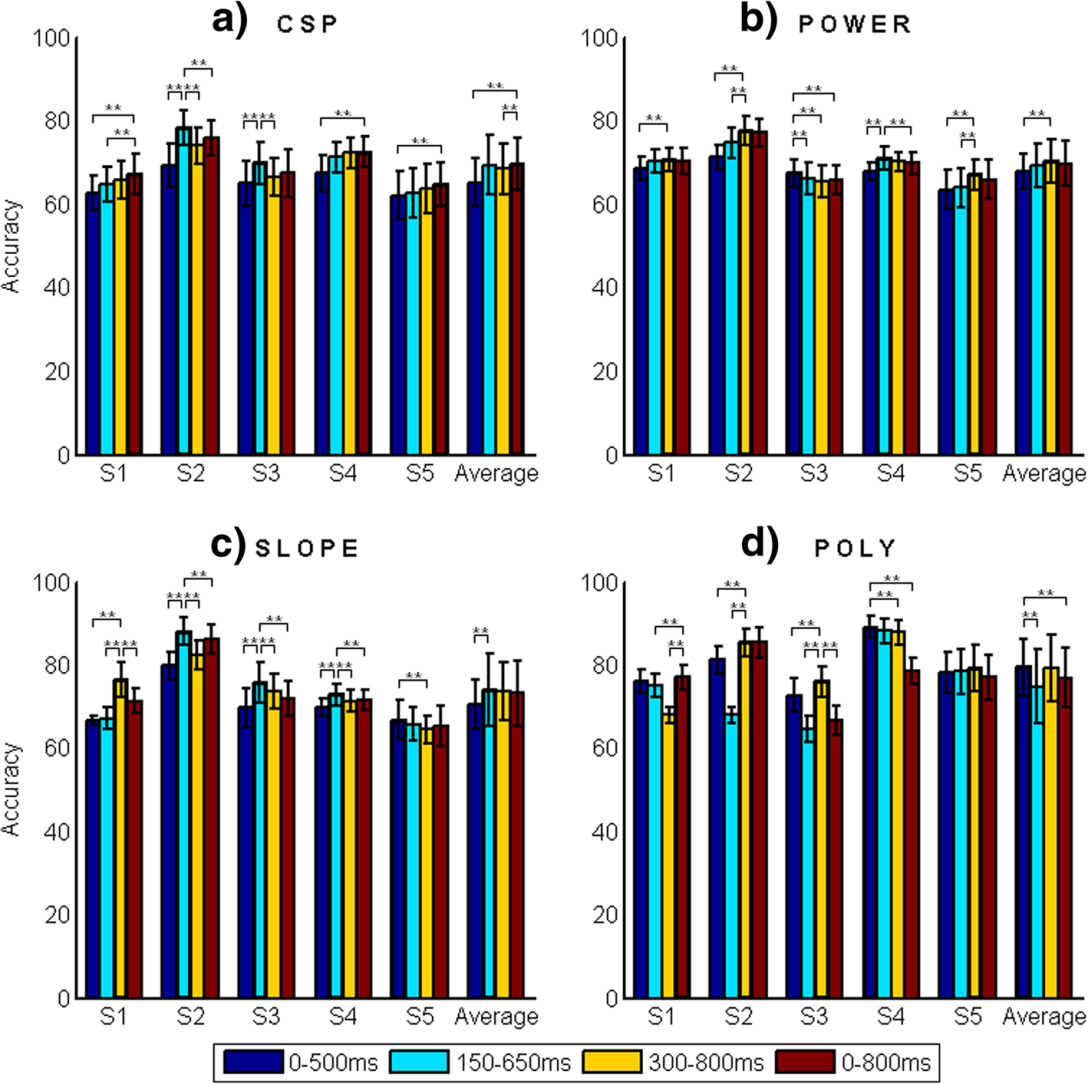
Accuracy achieved for each subject considering different features and different time intervals. **a** CSP, **b** Power, **c** Slope, **d** Poly

Under these conditions, a LDA classifier has been used in the classification stage. Training data set consists of 60 *%* of the total trials from each class, so the evaluation data set is the remaining 40 *%* (e.g. for S1: 31 *Reaction* + 62 *Normal walking* trials form the training data set). In order to assure that the data assigned for the training and the evaluation set do not influence the classification process, a random subsampling validation test of 100 iterations is performed. Table 2 shows the mean and standard deviation of the accuracy achieved for each subject with each of the four features extracted from the different time intervals. The maximum accuracy value for each subject using each feature is marked in bold. These results are graphically represented in Fig. 6. The bar with the maximum accuracy value has thicker border. Also, the figure shows the results of the Wilcoxon rank sum test to compare the accuracy rate achieved with the different time intervals.

**Table 2:** Accuracy achieved for each subject with different features extracted at different time intervals

C S P						
Time interval	S1	S2	S3	S4	S5	Average
0–500 ms	62.7 ±4.1	69.3 ±5.3	65.0 ±5.3	67.5 ±3.6	62.4 ±5.9	65.3 ±5.7
150–650 ms	64.7 ±4.1	**78.2 ±4.2**	**69.9 ±5.1**	70.9 ±3.8	62.6 ±5.4	69.4 ±7.1
300–800 ms	65.8 ±4.5	74.0 ±4.3	66.4 ±4.5	72.1 ±4.0	64.2 ±5.6	68.4 ±6.1
0–800 ms	**67.1 ±4.9**	75.9 ±4.3	67.4 ±5.8	**72.5 ±3.8**	**64.7 ±5.7**	**69.5 ±6.3**
P O W E R						
Time interval	S1	S2	S3	S4	S5	Average
0–500 ms	68.6 ±2.7	71.3 ±3.0	67.4 ±3.3	67.9 ±1.9	64.8 ±4.0	67.8 ±4.1
150–650 ms	70.2 ±2.7	74.7 ±3.7	66.1 ±3.9	**70.8 ± 2.8**	64.1 ±4.7	69.2 ±5.2
300–800 ms	**70.7 ±2.9**	**77.5 ±3.6**	65.6 ±3.9	69.9 ±2.4	**67.0 ±4.1**	**70.2 ±5.3**
0–800 ms	70.3 ±3.1	77.1 ±3.4	65.8 ±3.6	69.9 ±2.3	65.8 ±4.5	69.8 ±5.4
S L O P E						
Time interval	S1	S2	S3	S4	S5	Average
0–500 ms	66.7 ±1.0	79.8 ±3.4	69.7 ±4.8	70.0 ±2.3	**67.4 ±4.4**	70.6 ±5.9
150–65 0ms	67.1 ±2.6	**88.0 ± 3.3**	**75.8 ± 4.9**	**72.4 ± 2.4**	65.5 ±4.9	**74.0 ± 8.7**
300–800 ms	**76.5 ±4.0**	82.4 ±3.4	73.8 ±4.2	70.9 ±2.8	65.2 ±3.1	73.7 ±6.8
0–800 ms	71.4 ±3.1	86.2 ±3.4	71.9 ±4.2	71.2 ±2.4	65.5 ±4.6	73.3 ±7.8
P O L Y N O M I A L						
Time interval	S1	S2	S3	S4	S5	Average
0–500 ms	76.0 ±2.8	81.2 ±3.4	72.7 ±4.0	**88.8 ±3.2**	79.3 ±5.4	**79.5 ±6.7**
150–650 ms	75.1 ±2.9	68.0 ±2.0	64.6 ±3.0	88.2 ±2.7	76.5 ±4.9	74.9 ±8.9
300–800 ms	68.0 ±1.9	**85.4 ±3.3**	**76.0 ±3.6**	87.9 ±2.8	**80.6 ±4.8**	79.3 ±7.9
0–800 ms	**77.0 ±3.1**	85.4 ±3.7	66.8 ±3.4	79.5 ±3.2	76.7 ±4.7	77.0 ±7.1

The maximum accuracy value for each subject is marked in bold

**Fig. 7:**
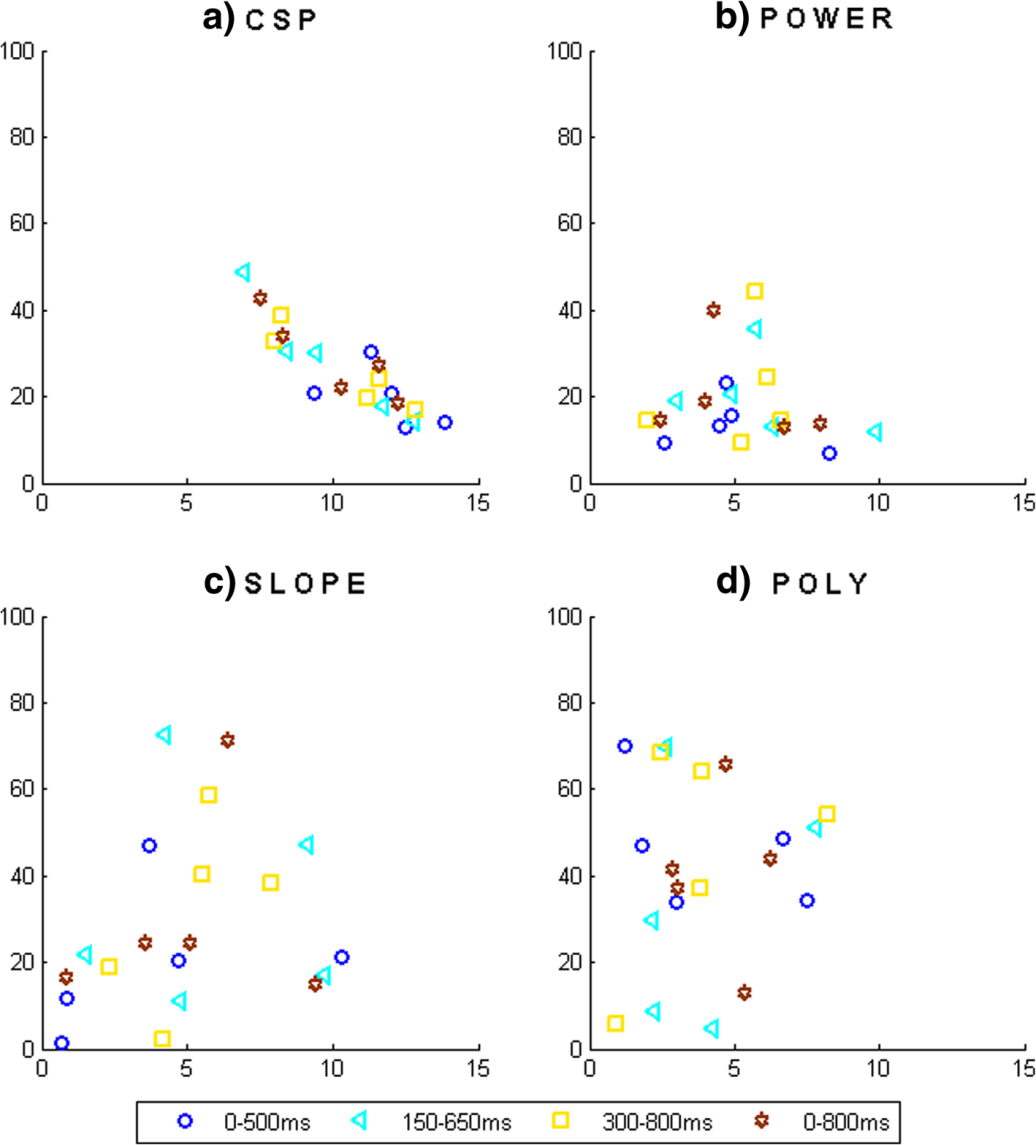
ROC space for LDA considering different features and different time intervals. **a** CSP, **b** Power, **c** Slope, **d** Poly. Each mark corresponds to one subject

Additionally, to make suitable comparisons of the performance achieved with different time intervals and select the best for each feature, it is important to assess the TP and FP rates. This behavior can be evaluated graphically in the Receiver Operating Characteristic (ROC) space [28]. Fig. 7 shows four graphics, each one containing the ROC space regarding to each feature. Each point corresponds to one subject at different time intervals. In order to improve the visualization, *x* axis is truncated until 15 %. Using all this information, it is possible to compare the classification results obtained with each feature extracted from the four time intervals and select the best one for each case.

For **CSP**, the maximum average accuracy is obtained considering the time interval 0–800 ms. A Wilcoxon rank sum test with significance level *α*=0.01 reveals significant differences between this value and those achieved considering the time intervals 0–500 and 300–800 ms. Analyzing the results obtained for each subject, S2 and S3 present the maximum accuracy considering the time interval 150–650 ms with significant difference regarding the remaining three. For S1, the maximum accuracy is reached considering the time interval 0–800 ms without significant difference to the accuracy obtained considering the time interval 300–800 ms. For subjects S4 and S5 the maximum accuracy is achieved considering the time interval 0–800 ms with significative difference to the value obtained considering the time interval 0–500 ms. Based on the results observed in the ROC space (Fig. 7a), the TP rate achieved considering the time interval 150–650 ms is higher than the obtained considering the time interval 0–800 ms in most of the subjects. Finally, FP rate stays low for all the time intervals. So, the selected time interval for CSP is 150–650 ms. Using the same reasoning, when the feature extracted is **power**, the optimal time interval is 300–800 ms.

In the **slope** case, the maximum accuracy for most of the subjects is achieved considering the time interval 150–650 ms. This interval also offers the maximum value in the average, with significative difference with the result achieved with the time interval 0–500 ms. Notice that the standard deviation with the time interval 150–650 ms is larger than in the other intervals. Moreover, exploring the ROC space (Fig. 7c) it can be seen that the performance obtained with the time interval 300–800 ms is more consistent than the obtained with the time interval 150–650 ms (in which the marks for all the subjects are more sparse on the ROC space). Therefore, the time interval selected is 300–800 ms. This fact reinforces the fact of analyzing the performance in the ROC space.

In the **polynomial** case, for most of the subjects the maximum accuracy is achieved with the time interval 300–800 ms. However, the maximum averaged value is obtained with the time interval 0–500 ms, without a significant difference compared to the accuracy achieved with the time interval 300–800 ms. The ROC space (Fig. 7d) shows that, although the time interval 300–800 ms offers higher TP rate for most of the subjects, the time interval 0–500 ms is more consistent for all the subjects, so, it has been selected for extracting the polynomial coefficients.

**Fig. 8:**
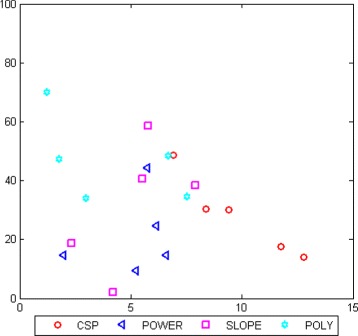
ROC space for LDA considering different features extracted in the optimal time interval. Each mark corresponds to one subject

With the optimal time interval selected for each features extraction algorithm, the next step is to compare which features offer the best classification. Figure 8 shows the ROC space with the results obtained for each subject with the four features extracted from their optimal time interval. As it can be seen, for most of the subjects, the polynomial coefficients achieved the lowest FP rate and a high TP rate (with a mean accuracy of 79.5 %). Slope feature also offers an acceptable performance, achieving a high TP rate for most of the subjects.

### Pseudo-online classification

To evaluate the performance of each feature in real time conditions, a pseudo-online classification is performed. The training set is made in the same way as described in the previous section (*Features evaluation*). The feature vector is extracted from the selected time interval obtained in the previous section using the recordings got during three runs. The fourth run is used as evaluation data. This recording is segmented into 500 ms windows, overlapped 400 ms, e.g. in an interval of 60 s, there are 596 windows, therefore 596 decisions must be performed. Under these conditions the pseudo-online classification is performed using each one of the features.

**Fig. 9:**
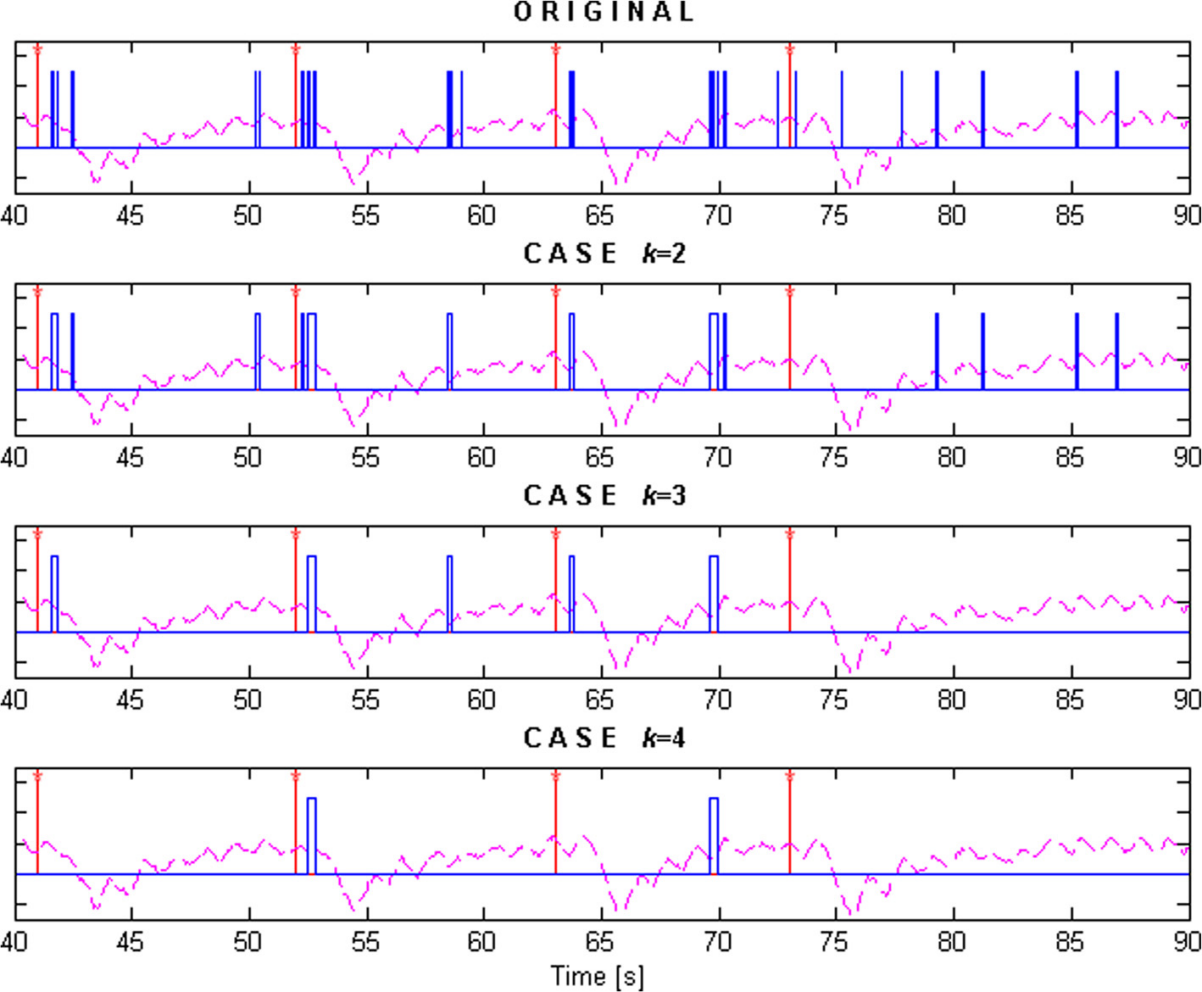
Reaction detections for different *k* values (2, 3, 4). The red line with asterisk is the instant when the obstacle appears. The blue lines are the reaction detections. The dashed magenta line represents the data recorded from the IMUs placed at the lumbar

In Fig. 9, the results of the detections for different *k* values (*k*=2,3,4) in a generic classification process are shown. The red line with asterisk represents the time instant when the obstacle appears. Each detection is represented by blue lines and the magenta dashed trace is the recording from the IMU located in the lumbar region, which helps to know the time instant when the subject physically reacts. The graphic on top corresponds to the results obtained from the classification without applying the reduction, while the detections for the different *k* values are shown at bottom. As it can be seen, when *k*=2, most reactions are correctly detected but a lot of FPs also appear. However, when *k*=4, the number of FPs decreases significantly, reducing slightly the number of true detections.

The results obtained for each subject with different *k* values are summarized in Table 3. Consistent with the previous section, the best results for most of the subjects are obtained when slope or polynomial coefficients are used as features. For the case of slope, it is noticeable that the FPs/min are reduced when *k* value increases not being the true detections so affected. For that reason, the optimal value of *k* is 4. In the case of polynomial coefficients, when the *k* value increases, the FPs/min are reduced, but the true detections rate decreases. Therefore, in this case the optimal *k* value is 2, achieving a satisfactory true detection rate (e.g., for S2 all obstacles are detected) with a maximum of 12.9 FPs/min.

**Table 3:** Pseudo-online results for each subject with features extracted at optimal time intervals

C S P								
Subject	Time recording [s]	No. Obstacles	True reaction detection			False detection/min		
			k =2	k =3	k =4	k =2	k =3	k =4
S1	186.0	14	4	3	2	18.88	13.63	9.01
S2	184.5	14	13	9	3	13.38	6.70	3.16
S3	184.0	14	2	2	1	5.65	1.77	0.71
S4	198.5	14	7	7	6	12.90	6.46	2.41
S5	115.5	6	4	3	2	19.70	15.06	8.80
P O W E R								
Subject	Time recording [s]	No. Obstacles	True reaction detection			False detection/min		
			k =2	k =3	k =4	k =2	k =3	k =4
S1	186.0	14	4	4	3	15.03	13.63	11.88
S2	184.5	14	12	12	12	19.36	17.25	15.85
S3	184.0	14	3	2	2	4.24	4.24	2.12
S4	198.5	14	6	4	4	9.96	9.06	6.64
S5	115.5	6	2	2	0	11.42	9.35	7.27
S L O P E								
Subject	Time recording [s]	No. Obstacles	True reaction detection			False detection/min		
			k =2	k =3	k =4	k =2	k =3	k =4
S1	186.0	14	6	4	4	22.73	12.94	8.04
S2	184.5	14	12	11	10	13.29	9.09	4.55
S3	184.0	14	4	3	2	3.88	3.53	2.12
S4	198.5	14	2	0	0	2.41	0.60	0.30
S5	115.5	6	2	2	0	4.15	1.55	1.02
P O L Y N O M I A L								
Subject	Time recording [s]	No. Obstacles	True obstacle detection			False detection/min		
			k =2	k =3	k =4	k =2	k =3	k =4
S1	186.0	14	5	4	4	12.94	10.48	8.04
S2	184.5	14	14	10	6	12.68	6.34	2.11
S3	184.0	14	6	1	0	8.48	4.94	1.77
S4	198.5	14	1	0	0	2.71	0.00	0.00
S5	115.5	6	4	1	1	11.94	3.63	1.55

## Discussion

Even though this study is oriented to be applied to the control of a lower limb exoskeleton, the results were obtained using data from healthy subjects. In [29] it was reported that there are not significant changes in the potential previous to movement (readiness potential) between subjects with spinal cord injuries and healthy subjects, and the potential which concerns us has similar nature. However, to assure that the potential does not present a significant change, an analysis of the brain activity of people with mobility disorders will be made.

As in our previous work [19], the results suggest that there is a change in the brain activity over the fronto-central area after an obstacle appears. These results are consistent with [18], despite avoiding the obstacle in a different way.

Moreover, it is important to remark the fact that the obstacle representation does not influence the behavior of the EEG signals, obtaining similar changes in the EEG signals for both obstacle representations. Therefore the obstacle detection by EEG signals is feasible since the change observed in the brain activity is related to the processing of the obstacle appearance and the preparation of the subject to react due to the unexpected obstacle, not depending of the way in which the obstacle is represented. This fact suggests that the behavior of the brain activity will be minimally modified with the appearance of a physical obstacle.

Other way to overcome the obstacle could be reducing the gait speed, applying in this case an analysis like in [17]. However, this approach has the drawback that the epochs employed to detect the change in the speed are large (4 s), and in this case, it is desirable to detect the intention to react in a reduced time interval in order to be able to send the control command to the exoskeleton.

As the change of the brain activity when the obstacle appears is noticeable in the time domain, selecting the optimal time interval for each feature is a transcending step since the features present different performance depending on the time interval used for their extraction. From the results, it can be seen that making a decision of the performance taking into account only accuracy values can sometimes lead to misleading results, so the relationship between TP and FP rates should be regarded to make an appropriate decision. In the case of CSP and power features, the utility of the ROC space evaluation is not noticeable. But in the case of the slope and polynomial features, it is crucial.

For the slope feature, the maximum accuracy for most subjects is obtained in the time interval 150–650 ms. However in the ROC space (Fig. 7c) the results achieved with this time interval for all subjects are very variables, as the points for all subjects are sparse in the plane. Conversely, the results obtained with the time interval 300–800 ms are more consistent for all subjects, which means that the points for all subjects are located close in the plane. This same behavior is observed for the polynomial coefficients, achieving the best accuracy for most subjects at the time interval 300–800 ms. However in the ROC space (Fig. 7d), it can be seen that the achieved results with the time interval 0–500 ms are more consistent for all subjects.

Once the optimal time interval is selected to extract each feature, the next step is to select the best feature. In Fig. 8 it is observed that the best results are obtained using polynomial coefficients, achieving a very low FP rate for most subjects and a higher TP rate than the obtained with the others features. Slope feature also provides an acceptable TP rate, and the FP rate is higher than the one achieved with the polynomial coefficients. Depending on the final application, based on this study, the researcher should decide how beneficial could be the FP rate reduction even if this reduction decreases the TP rate. Since the final application of this study is to send a command control to an exoskeleton to execute an emergency stop, it is preferable to obtain a high TP rate although FP rate would increase slightly. Under this reasoning, the best feature will be polynomial coefficients.

Despite the best time interval and feature were selected regarding all the subjects performance, this analysis could be carried out individually for each subject due to the variability of the EEG signals among individuals.

The reliability of the analysis is demonstrated through the pseudo-online classification, where the results are consistent with those obtained in the feature evaluation. That is, the best results are obtained with the slope or polynomial coefficients for most subjects. It is possible to see in Table 3 that for the best subject almost all obstacles are detected with a reduced FPs/min, e.g. using polynomial coefficients, 10 out of 14 obstacles were detected with 6.34 FPs/min. Although this is a reduced rate, in a real experiment it would mean that the exoskeleton would stop six or seven times per minute, being uncomfortable for the subject. In these conditions, the BCI system based on this processing would not be entirely satisfactory. More specifically, for subject S4, in spite of the fact that good results are obtained in the offline classification, the results achieved in the pseudo-online classification are not satisfying. Therefore, a priority step in our future work will be to improve the performance of the processing to reduce the FP rate. Even so, the reader must take into account that each minute 596 decisions are taken and these results are achieved with single trial, i.e., no average of several trials is calculated to enhance the signal to noise ratio, as it is common in the ERP detection. This is a remarkable observation towards a real-time application, where it is not possible to average several trials since the obstacle appears in a single time instant and with this information the BCI must be able to emit the control command.

Although the accuracy rates remains lower than 80 %, this first approach allows verifying that there is a characteristic pattern related to the change of the brain activity when an obstacle appears suddenly. Thus, it is feasible to detect the appearance of the obstacle with an accuracy higher than randomness before the subject reacts. This is an encouraging result considering that the final application is focused on controlling lower limb exoskeleton.

## Conclusions and future work

Through this paper different features of the EEG signals were evaluated in order to know which ones are the best to detect an obstacle that suddenly appears. The results suggest that it is feasible to detect the obstacle before the subject reacts. A first analysis showed that the best results are obtained using slope or polynomial features. These observations were consistent with those obtained in the pseudo-online classification.

In future works it will be necessary to evaluate if the EEG signals present similar behavior for people with mobility disorders in order to assure that there is not a significant change in the potential observed when an unexpected obstacle appears. Also new preprocessing and classifiers will be applied in order to improve the TP rate while the FP rate remains low. Furthermore, an online classification should be performed to test if the detection is achieved with the same performance as in the pseudo-online evaluation. After verifying this issue, the BCI implementing this procedure will be used to stop the exoskeleton during walking when an unexpected obstacle appears.
